# Amino acid sites related to the PB2 subunits of IDV affect polymerase activity

**DOI:** 10.1186/s12985-021-01703-z

**Published:** 2021-11-22

**Authors:** Yutian Wang, Weiyang Sun, Zhenfei Wang, Menglin Zhao, Xinghai Zhang, Yunyi Kong, Xuefeng Wang, Na Feng, Tiecheng Wang, Feihu Yan, Yongkun Zhao, Xianzhu Xia, Songtao Yang, Yuwei Gao

**Affiliations:** 1 Key Laboratory of Jilin Province for Zoonosis Prevention and Control, Changchun Veterinary Research Institute, Chinese Academy of Agricultural Sciences, Changchun, 130000 China; 2 Jilin Agricultural University, Changchun, 130118 China

**Keywords:** Influenza D virus, Viral polymerase, PB2 protein, Single-site mutation, Random mutation library, Mini-replicon reporter constructs

## Abstract

**Background:**

In 2011, a new influenza virus, named Influenza D Virus (IDV), was isolated from pigs, and then cattle, presenting influenza-like symptoms. IDV is one of the causative agents of Bovine Respiratory Disease (BRD), which causes high morbidity and mortality in feedlot cattle worldwide. To date, the molecular mechanisms of IDV pathogenicity are unknown. Recent IDV outbreaks in cattle, along with serological and genetic evidence of IDV infection in humans, have raised concerns regarding the zoonotic potential of this virus. Influenza virus polymerase is a determining factor of viral pathogenicity to mammals.

**Methods:**

Here we take a prospective approach to this question by creating a random mutation library about PB2 subunit of the IDV viral polymerase to test which amino acid point mutations will increase viral polymerase activity, leading to increased pathogenicity of the virus.

**Results:**

Our work shows some exact sites that could affect polymerase activities in influenza D viruses. For example, two single-site mutations, PB2-D533S and PB2-G603Y, can independently increase polymerase activity. The PB2-D533S mutation alone can increase the polymerase activity by 9.92 times, while the PB2-G603Y mutation increments the activity by 8.22 times.

**Conclusion:**

Taken together, our findings provide important insight into IDV replication fitness mediated by the PB2 protein, increasing our understanding of IDV replication and pathogenicity and facilitating future studies.

**Supplementary Information:**

The online version contains supplementary material available at 10.1186/s12985-021-01703-z.

## Introduction

Influenza viruses are currently the most common pathogens known to cause flu epidemics between humans and animals. Influenza viruses not only cause tremendous economic losses due to animal infection but can also directly infect humans despite the host barrier. In addition, reassortment events can occur with other flu viruses, even posing severe risks to human health. Influenza viruses belong to the family Orthomyxoviridae and comprise four species: A, B, C, and D. Influenza A viruses (IAVs) are known to cause occasional pandemics and seasonal epidemics. Aquatic birds are considered an important natural reservoir of influenza A viruses that can then infect various domestic fowls and mammals, such as horses, pigs, dogs, cats, tigers, and minks [1]. Influenza B viruses (IBVs) appear to naturally infect only humans and seals. IBV can simultaneously infect the upper and lower respiratory tracts and cause similar symptoms to type A [2]. Influenza C viruses (ICVs) cause upper respiratory tract infections among children, generally with milder symptoms [3].

Influenza D viruses (IDVs) were first isolated from swine suspected of influenza in 2011, but they have been found in cattle worldwide since 2014. The main reservoirs of IDV are cattle, swine, and other small ruminants [4]. IDV is similar to ICV in regards to structure and genes. So far, IDV is mainly divided into three pedigrees: D/OK, D/660, and D/Japan, and cross-reactivity is found among those three pedigrees [5]. Reports showed that IDV is more likely to mutate and evolve than ICV; hence, a continuous monitor for IDV variation is needed [6]. Serological investigation indicates that IDV could infect humans, as screenings showed that 1.3% of general populations presented anti-IDV hemagglutination inhibitory antibodies in serum [4]. When the positive rate of anti-IDV antibodies was analyzed in people who were professionally exposed to cattle in Florida, the seroprevalence of people raising cattle was as high as 95%, and the seroprevalence of individuals who had never been exposed to cattle could be as high as 18% [7]. It is still unclear whether the IDV viruses could cause human disease and whether there is transmission among humans. IDV infections have also been reported outside the U.S., including France, Italy, Ireland, Japan, China, Morocco, Benin, Togo, and Kenya.

Since the beginning of the twentieth century, four global influenza pandemics have been recorded: the so-called Spanish flu epidemic of 1918, the Asian flu pandemic of 1957, the Hong Kong flu of 1968, and the swine flu pandemic of 2009. Of these, the Spanish flu epidemic was the most severe one [8]. It is believed that the trigger for influenza pandemics is that the influenza viruses acquired a new type of hemagglutinin during the spread in the general population that lacks antibodies against this new hemagglutinin protein. Describing how the viruses adapt to new hosts will contribute to our understanding of the evolution and spreading of zoonotic infectious diseases that might eventually evolve into a human pandemic [9].

The influenza viruses polymerase is a key decision factor in hosting a range of viruses. The polymerase performs the transcription and replication of the virus genome. Mutations in polymerase could increase the viral pathogenicity towards mammals by enhancing the activities of viral polymerase or some other pathways. Multiple amino acid mutations in IAV polymerase proteins, such as PB2-E627K, are associated with high pathogenicity in mammals [10]. Substituting PB2-E627K can not only enhance the activity of polymerase proteins in mammalian and human cells but also contribute to the replication of influenza viruses in these cells. These two biological characteristics are closely related to the high pathogenicity of influenza viruses in mammals. However, there is a surprising lack of the E627K mutation in the 2009 H1N1 pandemic virus lineage. Instead, mutations at positions 590 and 591 of PB2 were detected, increasing polymerase activity [11].

As of now, researchers have used two methods to accurately understand the occurrence of IAV pathogenic enhancement: examining the changes that have occurred on influenza virus in the past and monitoring the evolution of influenza virus cultivation in human cells in the laboratory. However, these methods can only observe a small part of the many possible changes occurring in viruses at the genetic level and may neglect extra mutation sites and possible evolutionary pathways for zoonoses in the future. To overcome this potential problem, Soh et al. [12] mutated every single amino acid of influenza A PB2 one by one. Subsequently, they tested if the changes could help the virus grow better in human cells and enhance pathogenicity. In humans, IDV targeted antibodies have been found, especially in those individuals exposed to cattle for an extended period, indicating that the virus may infect humans. Moreover, the ferrets, which are good infectious animal models of the human influenza virus, are also susceptible [13]. It can be used to predict the possibility that IDV may have the ability to invade the lower respiratory tract, resulting in more severe symptoms. Unlike ICV, IDV can replicate well in cell cultures at 37 °C, which is the exact temperature of human lungs. Similarly, the HEF of IDV has a more open receptor binding region than that of ICV, indicating that IDV has a wider range of cell tropism [14]. Therefore, IDV may infect the lower respiratory tract of humans only via some adaptive genetic variations.

In this study, we created a random mutation library of the PB2 IDV viral polymerase subunit to test which amino acid point mutations in influenza D viruses will increase viral polymerase activity, leading to increased pathogenicity. Though with luck, the influenza D virus has not spread widely to cause a pandemic. However, understanding the changes in the polymerase of the influenza D virus can help to learn the increased pathogenicity of the virus and its adaptation to new hosts, which could be an essential factor that scientists need to consider when assessing the risk in future epidemics.

## Materials and methods

### Cell culture

HEK 293T-cells were maintained in D10 media (DMEM supplemented with 10% fetal bovine serum, 2 mM l-glutamine, 100 U/mL penicillin, and 100 mg/mL of streptomycin). All cell cultures were maintained at 37 °C in a humidified incubator with 5% CO_2_. The cell lines tested negative for mycoplasma.

### Plasmid construction

The pCAGGS vector used in the experiment was stored in the laboratory. The sequence of PB2 (serial number: JQ922305), PB1 (serial number: JQ922306), P3 (serial number: JQ922307), and NP (serial number: JQ922309) of the first influenza D virus D/Swine/Oklahoma/1334/2011 published on Genbank, were sent to Anhui General Biosystems Co., Ltd. for gene synthesis. Oligo 6.0 and Primer 5.0 software were applied to design the specific primers for 4 gene segments PB2, PB1, P3, and NP of IDV. The primers were sent to Changchun Comatebio Biological Co., Ltd. for synthesis.

For the generation of minireplicon reporter constructs for each of the four genomic segments of IDV, the synthesized gene plasmid was used as the template, using specific primers amplified those four fragments with Super-Fidelity Phusion DNA polymerase. The PCR process was carried out following the instructions of the Phusion enzyme, with the following cycle profile: 8 °C 10 s, 55 °C 20 s, 72 °C 90 s, for 30 cycles. Promega's DNA Gel Extraction Kit was used to recover 1% agarose gel electrophoresis target bands of PB2, PB1, P3, and NP. The four recovered PCR products and the pCAGGS vector were digested with NEB BsmB Ι enzyme, and the purified materials were ligated (molar ratio insert-to-vector, 1:10). The transformed JM109 colonies carrying the plasmids were extracted and sent to Changchun Kumei Biological Co., Ltd. for sequencing and identification. Plasmids with correct sequence alignment were named pCAGGS-PB2, pCAGGS-PB1, pCAGGS-P3, and pCAGGS-NP.

Design point mutation primers and site-directed mutagenesis were performed at position 627 of pCAGGS-PB2 from E to K (pCAGGS-PB2-627K), position 701 from P to D (pCAGGS-PB2-701D), and position 701 from P to 701N (pCAGGS-PB2-701N).

### Construction of a random mutation library of the IDV PB2 gene

Degenerate Primers were synthesized, carry out gene amplification of the IDV PB2 gene and ligate into the pCAGGS vector. Colony PCR was used to screen for transformants, and the selected positive clones were sequenced for verification. Then, high-purity plasmid extraction was performed.

## Results

**Fig. 1:**
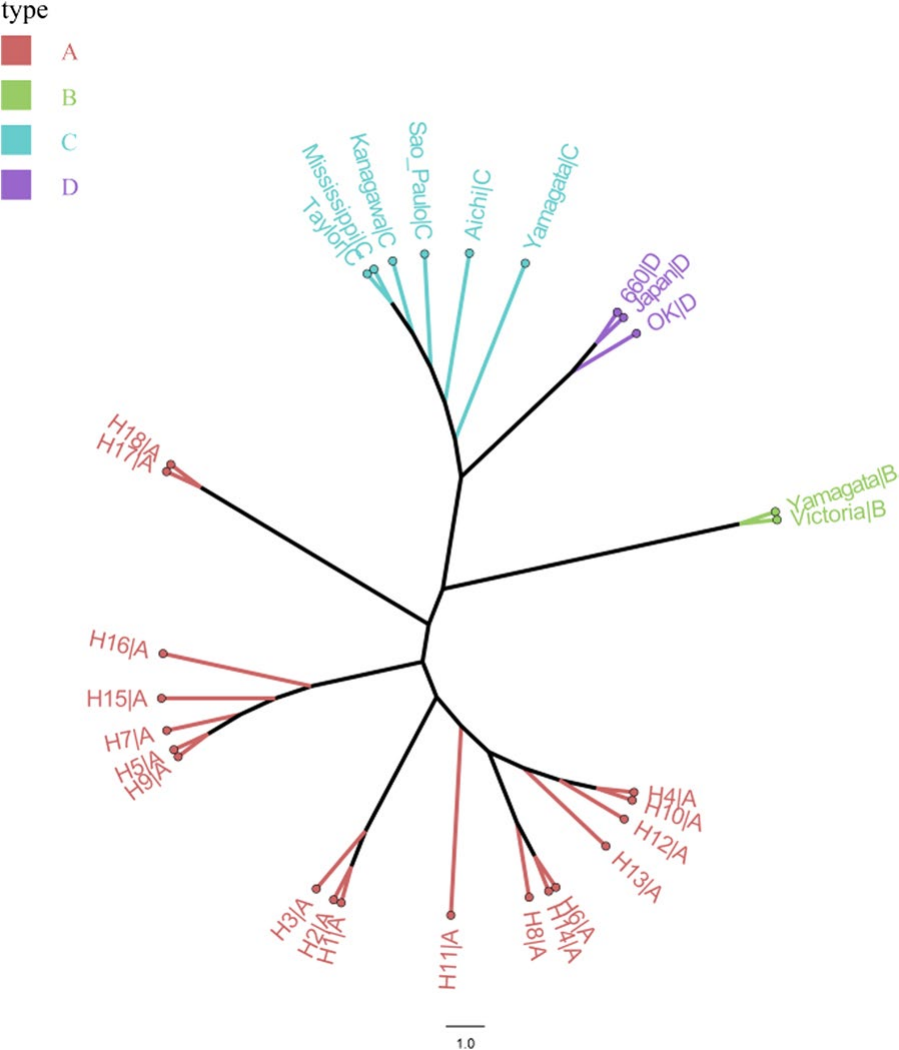
Phylogenetic tree of Influenza viruses based on PB2 sequences. Red branches on the phylogenies represent influenza A viruses, green branches represent influenza B viruses, blue branches represent influenza C viruses, and purple branches represent influenza D viruses. The black circle represents the strains in this study

The PB2 from the influenza D virus strain D/Swine/Oklahoma/1334/2011 (Hause et al., 2011) was selected. This strain was the first influenza D virus published on GenBank. PB2 from this strain is representative of PB2 from the influenza D virus (Fig. 1).

The cDNAs of the four genomic segments (PB2, PB1, P3, NP) of the influenza virus D/swine/Oklahoma/1314/2011 (D/OK) were amplified by PCR and cloned individually into the pCAGGS vector. The pCAGGS vector contained the human Pol I promoter and the murine Pol I terminator that directed the precise synthesis of vRNA from one strand. Plasmid construction of pPolI-Luci-NP-D employed seamless cloning technologies, and the plasmid contained the segment-specific non-coding regions of the NP gene and the transcription segments of luciferase protein ORF (Additional file 1: Figure S1).

**Fig. 2:**
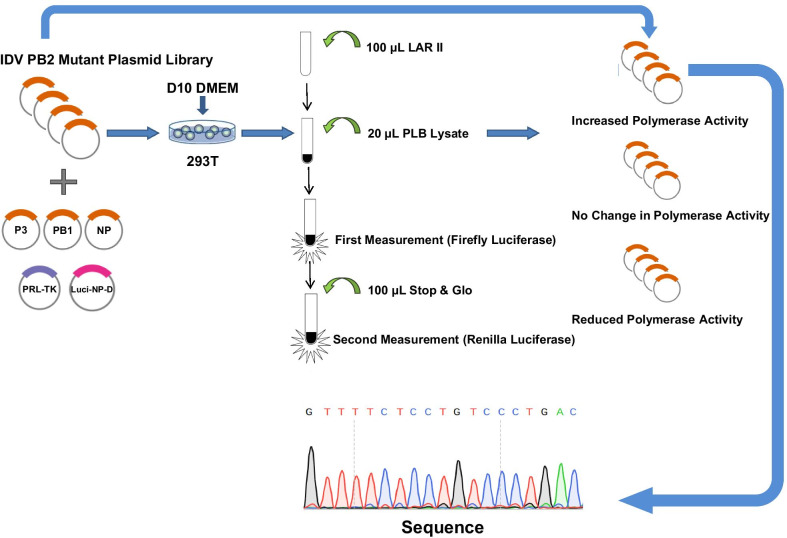
Construction of a random mutation library of the influenza D viral polymerase PB2 fragments. We mutagenized the 500–700 amino acid codon region of PB2 from an influenza D strain. Screening of unique sites for sequencing through microgenome experiments

Six protein expression plasmids, including pCAGGS-PB2, pCAGGS-PB1, pCAGGS-P3 and pCAGGS-NP, pPolI-Luci-NP-D, and the internal reference plasmid pRL-TK were used in the Microbial genomics experiment. These six plasmids were co-transfected in 293T cells to express the polymerase complex that can be combined with influenza-like vRNA, which was transcribed by pPolI-Luci-NP-D to form influenza-like vRNPs and expressed the firefly luciferase. Luciferin was used as a substrate to detect firefly luciferase, and coelenterazine was used as another substrate to detect Renilla luciferase. When the substrate of the renilla luciferase was added, the inhibitor of firefly luciferase was also added at the same time to interrupt the luminescence of Luciferin. So, only renilla luciferase activities could be tested during follow-up detection, achieving the goal with dual-luciferase reporter gene assay (Fig. 2).

**Fig. 3:**
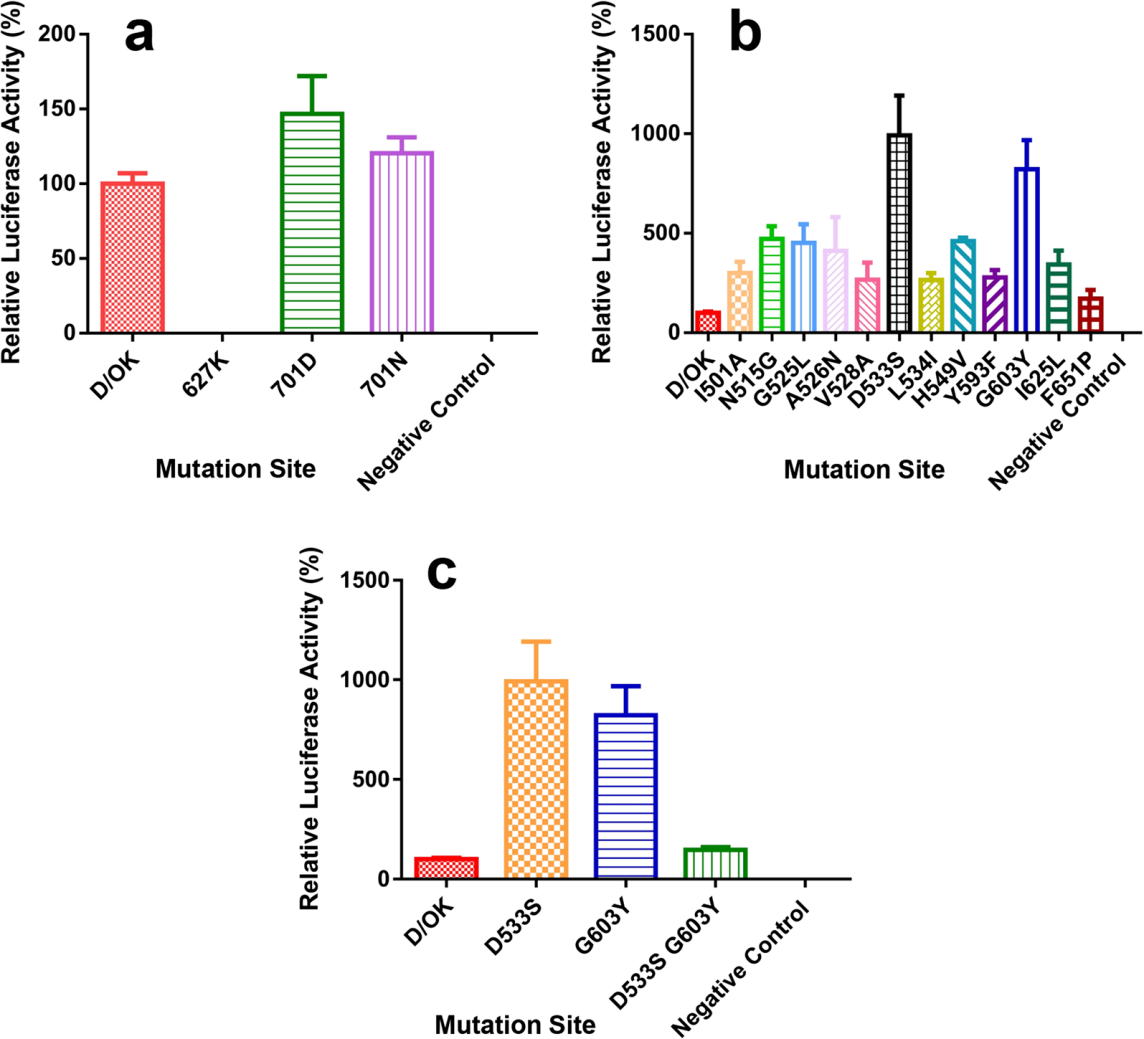
Viral polymerase activity. **a** Polymerase activity at key positions 627 and 701. **b** Partial site polymerase activity in mutation library. **c** PB2 D533S and G603Y double mutation polymerase activity

In order to confirm whether the changes of key sites 627 and 701 in PB2 could greatly impact influenza D viruses, we designed the point mutation primers to achieve the following mutations: E627K, P701D, and P701N. At key sites 627 and 701, the activity of IDV polymerase was significantly reduced in the E627K mutant compared to the wild type, indicating that the E627K mutation could not enhance viral replication. When it came to the P701D mutation, the IDV polymerase activity increased by 1.47 times, and the P701N mutation could also increase the IDV polymerase activity by 1.20 times (Fig. 3a).

A library of the PB2 gene was constructed by scanning random mutagenesis in the influenza D virus. This library was used to evaluate polymerase complex activities when the amino acid codons changed in a location range of 500–700 in PB2. We mutagenized 500–700 codons in PB2 to create a replicate mutant plasmid library. Some exact sites that increased polymerase activities in influenza D viruses are shown in Additional file 1: Table S1.

Both the single-site mutation of PB2-D533S and PB2-G603Y could increase the activity of IDV polymerase independently. The PB2-D533S mutation alone could increase the polymerase activity by 9.92 times, and the PB2-G603Y mutation alone increased the polymerase activity by 8.22 times (Fig. 3b). In addition to testing the effects of independent mutation on viral polymerase activity, the effects of two sites mutation combinations on polymerase activity were also tested. The test results showed that the two-point combined mutation of PB2-D533S and PB2-G603Y could increase the viral polymerase activity by 1.46 times, which was far lower when compared to the viral polymerase activity increased by these two mutations separately. This may be caused by unknown changes in the viral polymerase structure that result in a down-regulation of the efficiency of enhancing the viral polymerase activity when the two points mutated simultaneously (Fig. 3c).

## Discussion

Although the influenza D virus has not been isolated from humans, a cross-sectional serological study was performed on human serum samples from 35 cattle-exposed and 11 non-cattle-exposed adults to detect IDV antibodies using Hemagglutination Inhibition (HI) and Microneutralization (MN) assays. Overall, 94% of the cattle-exposed persons had a positive HI and MN titer. Characteristics of seropositive cattle-exposed persons included: 41% worked with sick cattle, 4% worked with swine diagnosed with respiratory illness, and 41% self-reported febrile illness in the previous year. When testing the people living close to cattle farms (with potential risk for infection), 8 of 741 serum samples collected were positive for HI antibodies (titers ≥ 40) [7].

Song et al. have analyzed the biological characteristics of the major surface glycoproteins in both IAV and IDV [14]. It was found that IAV can specifically bind to the receptors of *N*-Acetylneuraminyl-(2-3)-Galactose (originating in poultry) or *N*-Acetylneuraminyl-(2-6)-Galactose (human), and some subtypes of IAV can bind to both receptors. The bovine-derived IDV receptors are *N*-Acetylneuraminyl-(2-3)-Galactose and *N*-Acetylneuraminyl-(2-6)-Galactose, while the swine-derived IDV receptor is *N*-Acetylneuraminyl-(2-6)-Galactose, but not *N*-Acetylneuraminyl-(2-3)- Galactose. These findings suggest that the bovine-derived IDV may be transmitted between species just like IAV and even with more widespread cell tropism [15]. At the same time, the saccharide microarray experiment proves that IDV and ICV are similar in structure. The three-dimensional crystal structure of the HEF protein receptor complex of IDV shows the segments close to the top of HEF1, together with the 230-helix, 270-ring, 170-ring. In addition, unlike in ICV, three amino acids (T239, K235, D269) form a shallow cavity with an open channel, which may be one of the reasons why IDV gets a broader host spectrum [16, 17]. Although deadly infections have not been reported in humans, IDV significance in public health should not be neglected.

We can predict the key sites that may influence interspecies transmission by building a random mutation library of the IDV PB2 protein and screening the unique mutation sites. This study used amino acid point mutations at positions 627 and 701 of influenza D viruses. There are two positions in the PB2 protein that affect the growth of influenza A viruses in mammalian cells: the amino acid at position 627 (PB2-627) and the amino acid at position 701 (PB2-701) [10]. Several credible studies have found that there is always an E627K mutation of PB2 in clinical strains isolated from people who had died or were severely infected with specific subtypes of influenza viruses. Furthermore, this phenomenon occurred not only in one influenza subtype, but the PB2-E627K mutation can also be found in multiple isolates from the patients who were seriously infected or died from influenza, including H5N1 H7N7, H7N9, H9N2, and H10N8 subtypes. Hence, the PB2-E627K mutation may be closely related to the high pathogenicity to humans in these influenza subtypes [18–21]. Mutation D701N in the PB2 protein has been found in human influenza virus subtype H3N2, highly pathogenic avian influenza virus subtype H5N1, and highly pathogenic avian influenza virus subtype H7N7, which has also been verified to enhance the pathogenicity of influenza virus in mice [22–24]. Such mutation can enhance the virus replication capacity in mammals by increasing the viral polymerase activity [25]. We decided to focus on amino acids at positions 627 and 701 in influenza D viruses based on these previous studies. We found that in IDV PB2, mutations of the position 627E and 701P are not the key sites that could affect the activities of influenza D virus polymerase.

Based on the results of the amino acid positions at 627 and 701 of IDV PB2, we determined some other amino acids in the IDV PB2 protein and analyzed their effects on polymerase activity. The results have been classified into three sections. First, mutations at specific amino acid sites lead to a reduction in viral polymerase activity or even a loss of the expression capacity of the polymerase protein. Secondly, mutations at some specific amino acid sites show no effect on viral polymerase activity. Thirdly, mutations at specific amino acid sites contribute to increasing the activities of the viral polymerase. The statistical analysis found three amino acid sites (M532, D533, and L534) that had enormous implications for IDV polymerase activities. Mutations M532I, D533S, and L534I increased the polymerase activities by 3.26 times, 9.92 times, and 2.66 times, respectively, indicating that these positions are critical. In addition, mutations at the positions 515–519, such as N515G, D516E, and E519Y, increased the polymerase activities by 4.73 times, 2.26 times, and 2.33 times, respectively. Those positions should be considered and focused on the surveillance efforts of influenza D viruses. Moreover, the screening and characterization of other amino acid sites are still under investigation.

**Fig. 4:**
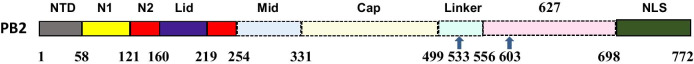
Schematic diagrams of the domain architecture of the PB2 subunit. The unresolved PB2-CTD is shown as transparent with dashed outlines

Through the study of PB2 protein structure elucidation, residues 256–689 of the PB2 subunit (the PB2-CTD), which comprise the mid, cap-binding, and 627 domains, are highly flexible and could not be resolved in either the apo or promoter-bound states. The two key sites are respectively located in the linker domains and 627 domains of the PB2 protein (Fig. 4), which are too flexible to be elucidated [26]. It makes us think more about the existence of critical sites in the domains that we have not yet determined could affect the activity of influenza D virus polymerase, which may affect the characteristics of interspecies transmission.

As a consequence, our approach represents a strategy to evaluate the capacity of IDV replication and pathogenicity based on the polymerase activity. Accordingly, the results of this research contribute to understanding, assessing, and monitoring the risk of a potential IDV pandemic that may occur in the future. That is a way to identify which of those mutations under observation during the viral surveillance is more likely to cause a significant increase in virus replication, pathogenicity, and even transmission. There are, definitely, some possible additional factors, including environmental and epidemiological factors, etc., not captured in this study. The data on amino acid mutations in this study can be combined with other data revealing evolution to explore the risks of IDV cross-species transmission.

## Conclusions

It can be concluded that the E627K mutation in influenza D virus resulted in a significant decrease in viral polymerase activity, while the P701D and P701N mutation would lead to a slight increase in viral polymerase activity. As a result of IDV PB2 protein random mutation library screening, it is found that the PB2-D533S mutation alone could increase the polymerase activity by 9.92 times, and the PB2-G603Y mutation alone could increase the polymerase activity by 8.22 times. Furthermore, the screening and characterization on other amino acid sites of IDV PB2 are still under researching. Our findings are valuable in helping provide important insight into IDV replication fitness mediated by the PB2 protein, facilitate the understanding of IDV replication and pathogenicity in future studies.

## Supplementary Information

**Additional file 1.** A schematic of Plasmid constructions.

## Data Availability

All data generated or analysed during this study are included in this published article and its supplementary information files.

## Abbreviations

IDV: Influenza D virus
BRD: Bovine respiratory disease
IAV: Influenza A virus
IBV: Influenza B virus
ICV: Influenza C virus
PB2: Polymerase basic protein 2
PB1: Polymerase basic protein 1
P3: Polymerase protein 3
NP: Nucleoprotein
HEF: Hemagglutinin-esterase
RT-PCR: Reverse transcription-PCR
ORF: Open reading frame
HEK 293T: Human embryonic kidney cells
vRNP: Virus ribonucleoprotein
HI: Hemagglutination inhibition
MN: Microneutralization
PB2-CTD: PB2-C-terminal domain
DMEM: Dulbecco’s modified eagle’s medium
RLA: Relative luciferase activity
FL: Firefly luciferase
RL: Renilla luciferase

## References

[1] Yoon SW, Webby RJ, Webster RG. Evolution and ecology of influenza A viruses. In: Compans R, Oldstone M, editors. Current Topics in microbiology and immunology - volume I. Springer: Cham; 2014. p. 359–375. 10.1007/82_2014_396. 10.1007/82_2014_396 24990620

[2] Li WC, Shih SR, Huang YC (2008). Clinical and genetic characterization of severe influenza B-associated diseases during an outbreak in Taiwan. J Clin Virol.

[3] Matsuzaki Y, Katsushima N, Nagai Y (2006). Clinical features of influenza C virus infection in children. J Infect Dis.

[4] Hause BM, Collin EA, Liu R, et al. Characterization of a novel influenza virus in cattle and swine: proposal for a new genus in the orthomyxoviridae family. mBio. 2014;5(2):e00031–14. 10.1128/mBio.00031-14. 10.1128/mBio.00031-14 PMC3958797 24595369

[5] Chiapponi C, Faccini S, Fusaro A (2019). Detection of a new genetic cluster of influenza D virus in Italian cattle. Viruses.

[6] Gatherer D. Tempo and mode in the molecular evolution of influenza C. PLoS Curr. 2010;2:RRN1199. 10.1371/currents.RRN1199. 10.1371/currents.RRN1199 PMC2995033 21127722

[7] White SK, Ma WJ, McDaniel CJ (2016). Serologic evidence of exposure to influenza D virus among persons with occupational contact with cattle. J Clin Virol.

[8] Mamelund SE (2011). Geography may explain adult mortality from the 1918–20 influenza pandemic. Epidemics.

[9] Herfst S, Schrauwen EJA, Linster M (2012). Airborne transmission of influenza A/H5N1 virus between ferrets. Science.

[10] Le QM, Sakai-Tagawa Y, Ozawa M (2009). Selection of H5N1 influenza virus PB2 during replication in humans. J Virol.

[11] Mehle A, Doudna JA (2009). Adaptive strategies of the influenza virus polymerase for replication in humans. PNAS.

[12] Soh YS, Moncla LH, Eguia R, et al. Comprehensive mapping of adaptation of the avian influenza polymerase protein PB2 to humans. eLife. 2019;8:e45079. 10.7554/eLife.45079. 10.7554/eLife.45079 PMC6491042 31038123

[13] Ferguson L, Olivier AK, Genova S (2016). Pathogenesis of influenza D virus in cattle. J Virol.

[14] Song H, Qi JX, Khedri Z (2016). An open receptor-binding cavity of hemagglutinin-esterase-fusion glycoprotein from newly-identified influenza D virus: basis for its broad cell tropism. PLoS Pathog.

[15] Peng GQ, Sun DW, Rajashankar KR (2011). Crystal structure of mouse coronavirus receptor-binding domain complexed with its murine receptor. PNAS.

[16] Martin LT, Verhagen A, Varki A (2003). Recombinant influenza C hemagglutinin-esterase as a probe for sialic acid 9-O-acetylation. Methods Enzymol.

[17] Chen Y, Liang W, Yang S (2013). Human infections with the emerging avian influenza A H7N9 virus from wet market poultry: clinical analysis and characterisation of viral genome. Lancet.

[18] Chen HY, Yuan H, Gao RB (2014). Clinical and epidemiological characteristics of a fatal case of avian influenza A H10N8 virus infection: a descriptive study. Lancet.

[19] Peiris M, Yuen KY, Leung CW (1999). Human infection with influenza H9N2. Lancet.

[20] Fouchier RAM, Schneeberger P, Rozendaal FW (2004). Avian influenza A virus (H7N7) associated with human conjunctivitis and a fatal case of acute respiratory distress syndrome. PNAS.

[21] Jong MDD, Simmons CP, Thanh TT (2006). Fatal outcome of human influenza A (H5N1) is associated with high viral load and hypercytokinemia. Nat Med.

[22] Jonges M, Bataille A, Enserink R (2011). Comparative analysis of avian influenza virus diversity in poultry and humans during a highly pathogenic avian influenza A (H7N7) virus outbreak. J Virol.

[23] Londt BZ, Brookes SM, Nash BJ (2013). Enhanced infectivity of H5N1 highly pathogenic avian influenza (HPAI) virus in pig ex vivo respiratory tract organ cultures following adaptation by in vitro passage. Virus Res.

[24] Czudai-Matwich V, Otte A, Matrosovich M (2014). PB2 mutations D701N and S714R promote adaptation of an influenza H5N1 virus to a mammalian host. J Virol.

[25] Jiao PR, Wei LM, Song YF (2014). D701N mutation in the PB2 protein contributes to the pathogenicity of H5N1 avian influenza viruses but not transmissibility in guinea pigs. Front Microbiol.

[26] Peng Q, Liu YQ, Peng RC (2019). Structural insight into RNA synthesis by influenza D polymerase. Nat Microbiol.

