# Supplementary material for: Amino acid sites related to the PB2 subunits of IDV affect polymerase activity
Source: Virol J. 2021 Nov 22;18:230. doi: 10.1186/s12985-021-01703-z (PMC8607657; doi:10.1186/s12985-021-01703-z)
Supplement: Supplementary file 1 — Additional file 1. A schematic of Plasmid constructions. [file 12985_2021_1703_MOESM1_ESM.docx]

Table S1. Result of some mutation sites with an impact on the polymerase activity

| Sample No. | Relative Luciferase Activity(%)* | Amino Acid Positions |
| --- | --- | --- |
| 1 | 326 | M532I |
| 2 | 279 | Y593F |
| 3 | 473 | N515G |
| 4 | 461 | H549V |
| 5 | 266 | L534I |
| 6 | 992 | D533S |
| 7 | 272 | F651P |
| 8 | 165 | N543A |
| 9 | 160 | E571L |
| 10 | 180 | L590F |
| 11 | 233 | E519Y |
| 12 | 163 | R588V |
| 13 | 226 | D516E |
| 14 | 452 | G525L |
| 15 | 822 | G603Y |
| 16 | 267 | V528A |
| 17 | 412 | A526N |
| 18 | 217 | K559I |
| 19 | 343 | I625L |
| 20 | 161 | V684F |
| 21 | 144 | D544F |
| 22 | 300 | I501A |
| 23 | 148 | N517P |
| 24 | 4.52 | S614H |
| 25 | 0.18 | E634V |
| 26 | 0.33 | E634S |
| 27 | 4.76 | G579Q |
| 28 | 0.22 | L512R |
| 29 | 0.06 | A617V |
| 30 | 0.02 | T669I |
| 31 | 0.16 | V694K |
| 32 | 0.68 | V545E |
| 33 | 0.40 | I691G |
| 34 | 0.34 | R618F |
| 35 | 0.26 | H549Q |

*$RLA (\%)=\left( \frac{FL}{RL}-\frac{Negative control of FL}{Negative control of RL} \right)\times100$


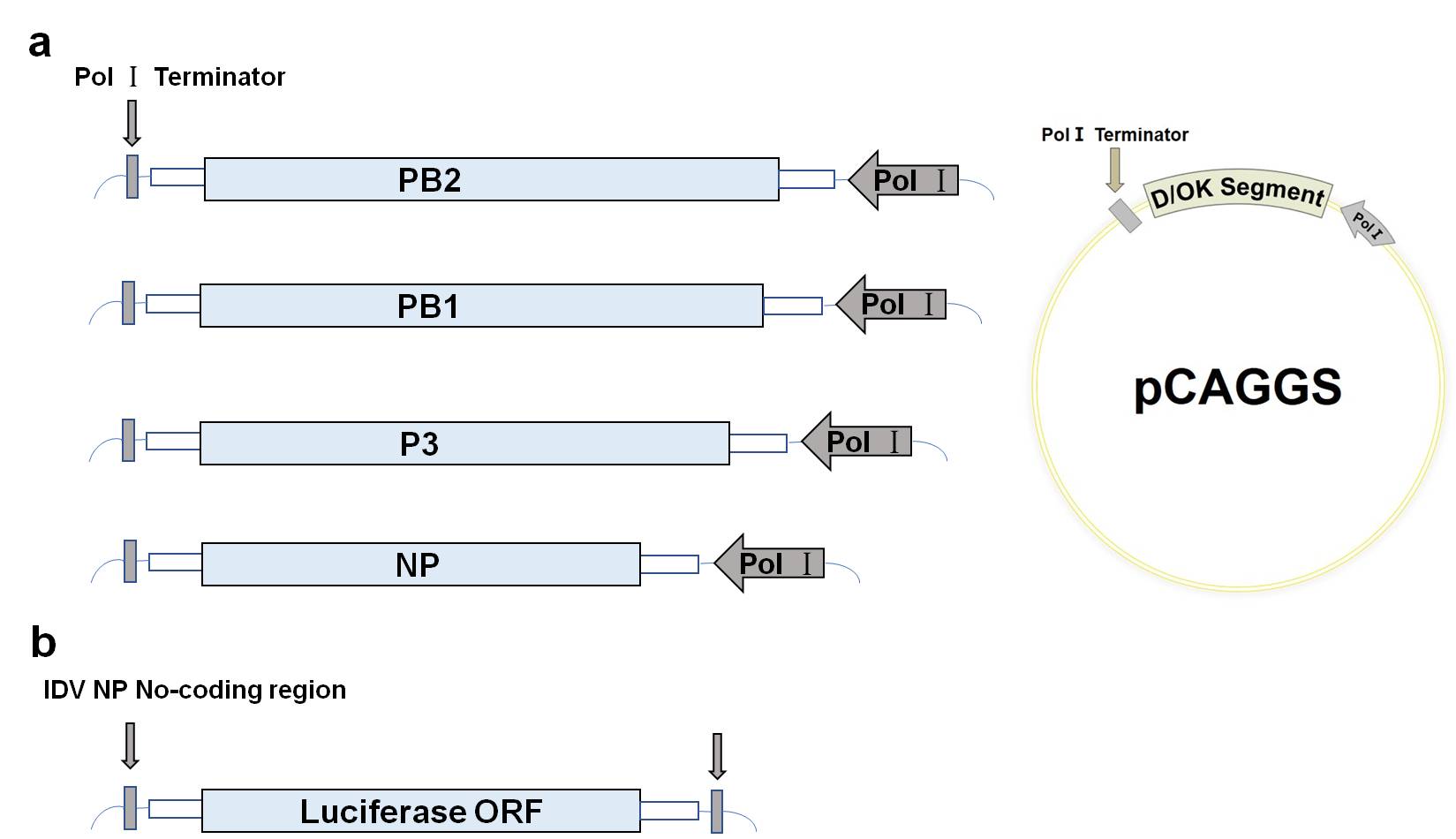


Fig.S1 (a) The four IDV segments were cloned into the pCAGGS vector. Each of the segments was flanked by the human Pol I promoter and the murine terminator. (b) The pPolI-Luci-NP-D plasmid contained the segment-specific non-coding regions of the NP gene and the transcription segments of luciferase protein ORF. The segment were cloned into the pCAGGS vector.
